# Selfhood and inclusive publics: a critical disability lens and creative practice to ground collaborative homelessness research

**DOI:** 10.3389/fsoc.2025.1591246

**Published:** 2025-10-02

**Authors:** Temba Middelmann

**Affiliations:** Independent Researcher, Toronto Metropolitan University, Toronto, ON, Canada

**Keywords:** homelessness, research, advocacy, critical disability, public space research, networks of homelessness service providers, homeless people, creative practice

## Abstract

This paper explores homelessness, research and advocacy with a critical disability lens. It reflects on several years of public space research, and advocacy in national and local networks of homelessness service providers, homeless people, and activists in Johannesburg, South Africa. The paper builds on a triad of self-(an)other-collective to unpack how sacrificing aspects of the self in conditions of extreme inequality and polarization is key for building broader collectives and inclusion. Reflecting on disability as a cause and consequence of homelessness, I offer insights from within and beyond research methods on everyday practices of community wellbeing. Firstly, at the level of community amongst public dwellers and interactions with individual service providers, and secondly through networks of practice-based organisations. The social model of disability clarifies systems of exclusion, de-pathologizing people living with homelessness, critical for expanding publics and inclusion. The paper explores moving towards collaborative and co-created research, guided by community and creative practice, drawing from the intersection of theory and practice around homelessness and disability. It also examines how the sustainability of collaboration also rests on internal shifts, which this paper explores through autoethnographic analysis of the author’s merging art and research practices. While practising public art has been valuable in building relationships in public space research, my art practice also aids in healing my own pyscho-spiritual self and bringing me more into community. The paper also follows Paula Toledo in centering curiosity as a basis for compassion and connection, key for substantive collaboration across difference, which also requires openness and honesty about complicity in conditions of inequality. It concludes by drawing out methodological implications from the intersection of these ideas, arguing for greater attention in research to time, play, creativity, openness about personal connection, and the importance of collaboration.

## 1 Introduction

One of the most striking absences in my research on Johannesburg’s inner-city public space was public toilets. Over the course of this research spanning my masters, PhD and first postdoctoral position between 2015 and 2022, I became increasingly aware of how the lack of public toilets was entangled with the way acceptable behaviour was understood and policed. Particularly, how homeless^1^ people were criminalised through being forced by circumstance to conduct private activities, such as their ablutions, in public spaces. This denial of access to public toilets in the face of circumstances (homelessness) that demand its necessity resonates with how many of the barriers faced by disabled people are based in their denial of access according to social structures and prejudice that shape forces of marginalization. Importantly, ways in which homeless people and disabled people are marginalized tend to obscure the skills, creativity, resilience and value of people, which allows prejudice to further take hold and exacerbate the same marginalization.

These forms of marginalization are linked to contexts of individualism, where many systems, especially in the neoliberal order, reward and acknowledge individual success at the same time as exacerbating injustices such as the impact of the housing crisis on homeless people, and the uneven impacts of climate change on marginalized people around the world (Bezgrebelna et al., 2023). Furthermore, homelessness as well as madness (or other forms of disability) are often presented under neoliberalism as the result of individual failure rather than structural conditions (Martin, 2022). This in turn is related to humanity’s fears and anxieties about scarcity, health, access, safety and survival, along with ideological polarization, which exacerbates an ‘us-and-them’ syndrome (e.g., Ling, 2023; Runswick-Cole, 2014). Because (social) research is conducted by people who bring aspects of themselves to their work, this paper explores personal circumstances and actions that can support shifts towards more collaborative, inter-disciplinary research based on more centring of lived experience (Martin, 2022). Critically, this centring must not preclude collaboration across difference.

This paper explores how internal shifts are important in moving towards collaboration, and how this ties research and life more closely together. This drives the key argument that mutually constitutive practices of wellbeing both rest on internal shifts towards others, as well as necessitating interdependence and collaboration across difference. I explore how my art practice, concurrent to my research work over the past decade, prompted and shaped internal shifts in my own life. At the same time, I touch on the resistance, solidarity, and communities of care I observed in the context of homelessness that also speak to the constitution of inclusive publics. In reflecting on a long period of research and practice, the paper is a theorisation of the connection between methods, ethics, and values in research over time. It aims to bring critical disability studies, creative practice, homelessness research and advocacy, into conversation, using autoethnography to reveal methodological insights for research aimed at community wellbeing.

An important dimension of my public space research and my art practice has been considering the relationships between individuals and collectives; the thresholds between selves and others. This paper draws on the ideas informing my entry into public space as a researcher, which began a long-term process where my praxis and theoretical endeavour shaped each other in a process of iterative feedback. Importantly, this iterative process brought me closer to the people and communities I was working with, constituting an entry into public space as a member of the public. This was shaped, and in turn played a role in shaping, my personal art practice. As such, the paper uses critical autoethnography (Maréchal, 2010) to trace my academic journey and it is merging over time with my artistic practice. It starts by exploring my entry-point as a historian into researching public space, and how this intersected with my collecting and painting work, ultimately shaping how I understand the research process and city around me (Middelmann, 2019, 2024). From there I examine my deepening research into public space and the threshold of publicness and privateness, between the self and other in space. This process also allowed me to recenter my appreciation for mystery and curiosity in the complex entanglements of urban space, finding further resonance between artistic, academic, and personal development. It is important to note that ‘clear and safe boundaries’ for protecting oneself and one’s energy are key for moving towards and with others (Cassina, 2022: 20).

This trajectory has taken me towards focus on the links between individual and collective wellbeing, especially in contexts of inequality and difference. This has fundamentally shaped my research practice towards greater collaboration, deepening my mutual use of public space with others while finding opportunities to work in community on issues facing people using these same public spaces. This connection of research with impact on real life issues is a shift occurring on wider scales (Brenninkmeijer, 2022), and here I explore the nature of this connection in my own work. This involves discussing the work that happens around the edges of our research, without being a central or official part of a particular research project. In other words, why do we choose to do the research that we do, and how does this relate to how we live our lives? This paper interrogates implications for research methods that stem from exploring one’s own connection to their research and its participants through a triad of self, other, and collective. Also, it explores how these are shaped by structural and systemic realities at different scales, including university employment and neoliberal capitalism. As such, the paper looks at debility (in the sense of how these contexts and circumstances are debilitating) *and* disability (in the sense of differences which have not been accommodated for, creating disability) (Puar, 2017), showing how these interrelate with homelessness, society and research.

## 2 Background: individualism and public life in research and art

Starting with an interest in public memory and undergraduate training in history, I began researching public space in Johannesburg at a human rights and heritage precinct named Constitution Hill in 2015. This expanded from 2017 to research a wider selection and variety of public places. These included a large park, a large open square and bus terminus, Constitution Hill, and a section of street and pavement that connected the three (Middelmann, 2020). The overarching research question was investigating the interplay between history, design, management and use of public space, with a spatial justice lens. The case study selection was aimed at having a wide variety of public space typologies (park, square, transport hub, heritage site, street, etc.), as well as choosing some of the largest and more significant public spaces in the inner city. Selection of research participants was done through iterative ethnographic research in the different spaces over the period 2015–2022, visiting each space regularly throughout, discussed further below. The author gradually moved from relatively organic and chance interactions to encounters guided more explicitly by emergent research questions. Ethnographic research was buttressed and triangulated by interviewing a wide variety of practitioners, public space users and managers, staff of relevant organisations, and experts, as well as archival research on the history, design and construction of the spaces. A key theoretical endeavour that started with this research, and is continued in this paper, is about exploring the shifting meanings of publicness and privateness, and also focusing on the threshold of interaction between public and private. I explore this threshold in the paper by looking at how individuals (private) interact with others and form collectives (public).

**Figure 1:**
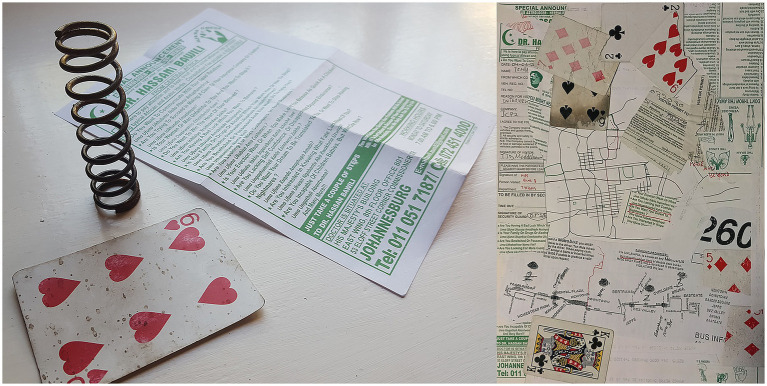
On the left is a collection from a day’s research in 2018 of playing card (the significance of playing cards, both discarded and collected, is reflected on in Middelmann (2024), which also discusses the links between collecting, research, and artistic production.), spring and ubiquitous Johannesburg-style pamphlet. On the right is a collage juxtaposing collected objects from research-fieldwork with author’s hand-drawn map of the research area.

During this period (2017–2020), I was exploring how my largely studio-based, visual art practices of painting and drawing were merging with my research practice, in turn becoming more public-facing and influenced by my relationship with public spaces and publics. Part of this was about bringing different parts of myself together, and also about bringing my life and work closer together, important for ethnographic public space research and my autoethnographic reflection (Morreira, 2012). I had been practicing urban collecting since childhood, fascinated by the objects discarded and lost in public space, but only during my PhD did I realise how this kind of observational, collecting and juxtaposition practice was part of my research on public space (Figure 1; see Middelmann, 2024). Important in the shift towards sharing my art, collaborating with others, and working in more public-facing ways, was starting to practice land art in public spaces with increasing regularity. Land art involves drawing and sculpting with found, natural, local materials; the practice of this art was formative for me in how I interacted with my surroundings, both human and non-human. This evokes Siebers’ concept of “complex embodiment,” described by Kim et al. (2021: 10) as ‘the reciprocal dynamic between environments and the human subjects who inhabit and create them.’ Crucial for my work was simply spending more time, and slower time (Piepzna-Samarasinha, 2019), being present in my surroundings, which facilitated deeper reflection on the nature of my relationships with people.^2^

**Figure 2:**
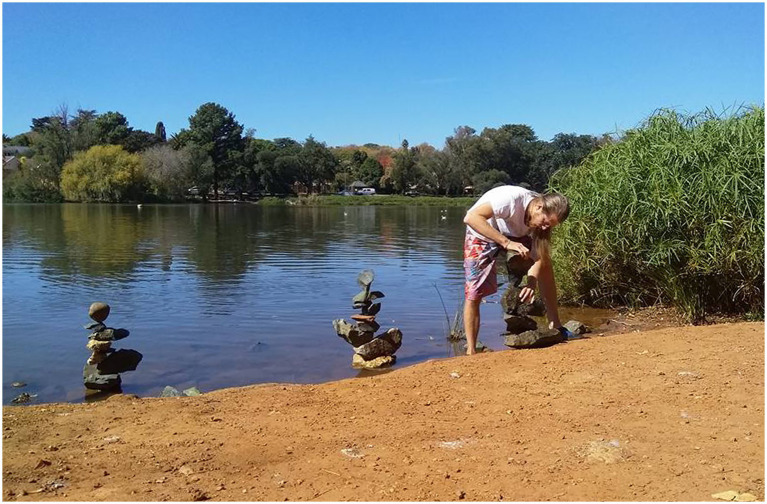
Author building stone balance sculptures in Emmarentia Gardens, Johannesburg 2018. Intentionally making land-art in spaces frequented by other people was part of my turn towards others as an artist *and* researcher, partly through sharing my authentic self through creative practice.

These shifts were important for my ethnographic immersion in the public spaces I was researching (Scholl et al., 2014), and also for my autoethnographic work which involved considering how my art practice had *already* been facing others by drawing on paper in semi-private spaces. By drawing and sculpting in public space through land art, it could turn further outward, which has expanded the range and nature of my social connections, partly by expanding my connection with non-human surroundings. Indeed, this type of practice has been one that also has brought me into a range of interactions with other humans in the spaces I make land art (e.g., Figure 2 below). This has been important to my art, research, and life, which I cannot disentangle. Indeed, in highlighting the importance of autoethnography in this work, I follow May (2023) who argues that the type of practice-based research I reflect on here can usefully incorporate autoethnography, especially in demonstrating entanglement between different aspects of practice with the body and mind doing the work. This kind of experimental, playful, creative presence in public space straddles the boundary between the idea of a researcher who leaves no trace and one who acknowledges that their presence and actions have significant impacts.

Both my art and research began as individualised practices, fairly common for both fields of work. In academia, this is necessitated by evaluation and impact measurements that value individual success, linked to requirements of obtaining degrees to build one’s individual profile to secure academic jobs; in needing to continue churning out journal publications, which supports competition more than collaboration (Riles, 2022; Kemp, 2013). This focus on individual brilliance in academia also comes with exclusion of women and minorities and is tied up with ableism: ‘the fascination with brilliance in philosophy and other areas could conceivably create an atmosphere in which displays of intellectual prowess are rewarded and imperfections are to be avoided at all costs’ (Cimpian and Leslie, 2017: 62). Where ways of being and thinking do not correspond with social norms shaped by academic departments dominated by white male leadership, narrow interests undermine inclusion (*ibid*.). However, collaboration by researchers with communities is growing, with calls for more focus on relationship building and less on metrics and publications (Lewis and Sadler, 2021). For artists, much is also dependent on one’s personal profile, with connotations of individual brilliance, unique talent, or even genius. Self-expression is often regarded as a core of artistic practice, despite there being many historical and current practises of art that are more communal, collectivist, and focused on social justice. These individualistic framings link with the ‘broad cultural logics of autonomy, self-sufficiency and independence’ that are denoted by ableism (Whitney et al., 2019: 1478), which also shape prejudiced attitudes towards homeless people, and contribute to conditions that undermine communities of wellbeing. This demonstrates the impacts of disability and disablism as well as highlighting debility, discussed further below.

For many years I was compelled by these notions of individual success, partly because these are still powerful in society, and partly because I am at an early stage of my career following the individualised task of attaining a PhD. Also, because of how societal circumstances tend toward placing responsibility on individuals for inculcating systemic change, regardless of circumstances of power (Argüelles et al., 2017). As such, individualism extends beyond sectors like art and research, connecting to discourses around wellbeing and wellness, the distinction between them demonstrating some tension in relationships between individuals and collectives (Mayson, 2021). Where wellbeing demonstrates a connection between individual and communal health, wellness is often targeted and marketed at individuals as part of capitalist entrepreneurialism. As Dorkatch (2024) has stated, ‘wellness culture today is radically individualised and it is kind of narcissistic and inward focused, and it leaves behind the broader systemic factors’. This paper explores the potential for individual shifts towards focusing on systemic factors as part of research and action towards collective solidarity and wellbeing. While critiquing responsibilization, the paper is wary of how such critiques can be used towards absolving ourselves of a reasonable level of responsibility towards collective issues (Mustalahti et al., 2020), which is also problematic.

I am in an ongoing process of trying to extricate myself from embeddedness in individualistic framings of success and wellbeing, notwithstanding various pressures and limits that affect the process. This involves reflecting on how my individualism, and moves away from it, have been shaped by and shaped my research methods and practice. It has been daunting to consider sacrificing some of my attempts to prove myself as an individual, concerned about what would happen if my contributions were ‘reduced’ to being an ‘*et al*’ or merely a contributor, potentially undermining my fantasy of being the author, creator, inventor, standing alone in my newly recognised brilliance. Yet as these shifts have found space to take root, I have been more deeply connected to others and at the same time more deeply connected to myself. This involves spending honest, reflexive time with oneself, and spending honest, open time with others, and allowing those processes to inform each other, which is where community is formed. Atkinson et al. (2024 unpaginated) further this by using the idea of crip time to explore ways of slowing down and resisting neoliberalism:

‘rest, recuperation and recovery time considers how we are thinking about ethical pacing and ways of working together. … to push the boundaries of what’s possible (or not) in the neoliberal academy and to play with the temporalities of normative research processes which are typically fast-paced and output-oriented.’

Accordingly, time is a key methodological issue that this paper draws out, arguing to protect the time it takes to develop genuine connections that can transcend the strictures of research agreements, funding deadlines, authorship negotiations. In places of deep division, simple presence can start connections across difference. Spending slow time in public spaces, at the pace of those living in or using those paces, connects crip time with the common community-based research refrain of ‘meeting people where they are’. As such, presence in public space was the beginning of an iterative approach to research methods, whether deliberate in terms of aiming to connect with the rhythms of regular users of each space, or more playful through creative practice and simply being myself in public space.

**Figure 3:**
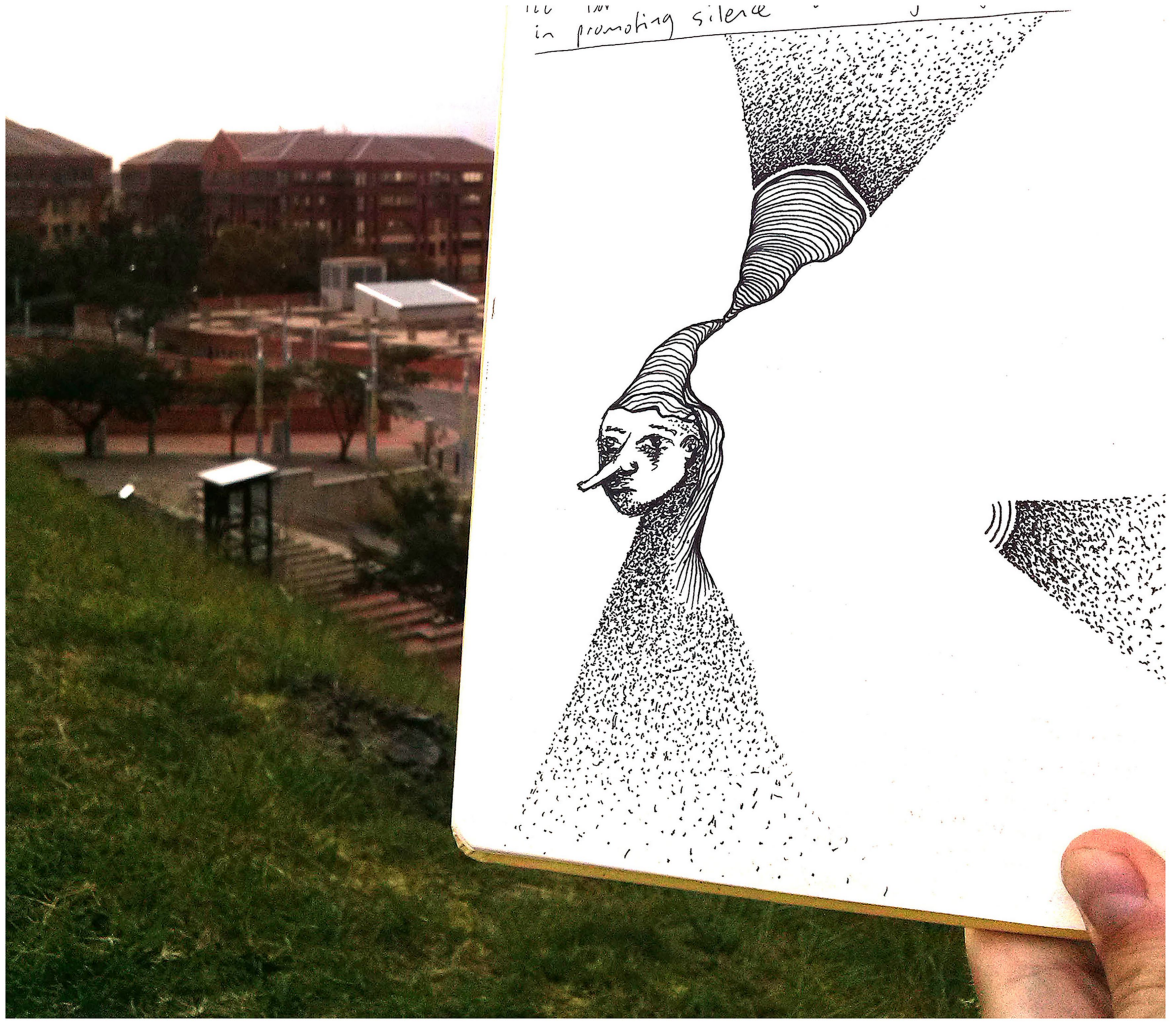
Drawing by author from 2016 during one of the first phases of public space fieldwork. The drawing evokes the early stages of becoming a member of the public, showing a disembodied self sending out signals to their surroundings. By being and knowing myself better in public space, I was working towards connecting with others. Sitting and drawing in public space as part of my research practice also evokes crip time—this was not active research in the sense of careful observation and intentional conversations/interviews—it was a case of an introverted, neurodivergent artist-researcher being and becoming themselves as an entry-point into the research process. A key connecting idea here is about cultivating authentic presence in public space, which I argue below is key to genuine connections and collaborative research and advocacy.

Practicing art in public space, i.e., playfully exploring my manner of presence (Stanford, 2016), was foundational to me finding ways to spend time in public space as a member of that public. The connection of play and art has been inspiring in finding ways to connect with others, and also with my inner-child-like sense of wonder, curiosity, and learning, which I think are important qualities for adults, especially seeking to practice inclusion (see Figure 3). Fearn (2022: 129) explores how this involves moving outwards towards others as well as inwards towards better knowledge of self:

‘play narratives blur the boundaries between human and other-than-human, providing evidence of the flow of communication and identification between children and other species. This supports a felt sense of connection and, exploration of sameness and difference engenders a sense of belonging and a gateway to enter the spiritual life’

Fearn (2022) (*ibid.*: 125) teases out the interplay between selves and others, public and private, that is always in flux; a ‘flow from inner to outer worlds and back again’ that this paper explores through the triad of self, other and collective discussed below. Without art, and as such play, I was often, like many other South Africans (and people in various parts of an increasingly polarized world), hamstrung by the tension of division and inequality in public space that often leads to a retreat into privacy, especially by white, relatively wealthy people like myself (Landman, 2019). As such, the internal shifts discussed in this paper were about finding my own belonging in a way that opened me into interaction with others. The way this relied on a merging of my art and research practices connects with how ‘political participation is increasingly intertwined with identity, self-expression and everyday life’ (Mahoney et al., 2021: 568).

As mentioned, partly driven by my position as a student pursuing research-based degrees, a lot of my work was both individualised and highly time-bound. I was struck by the constraints of this, though found little to mitigate it during my Master studies in 2015/6, with very limited time for fieldwork. In aiming to mitigate this and avoid abandoning emergent connections, I selected the same focus and area of study for my PhD, and subsequently for my first postdoctoral position. While this did not always mean I could actively slow down, there were ways in which this meant I could spend slower time in the spaces I was researching, and develop familiarity and connections with the people that used these spaces. As I built this familiarity with people and spaces, I could increasingly guide my interviews according to local realities, focused on balancing questions around history, design, use, management, with those focused on methods emerging from first phases of research, with those targeted at the interviewees specific area of expertise and/or experience. Analysis was similarly built up iteratively, with manual coding conducted according to key themes that emerged from each phase of fieldwork.

One of the key tensions in these public spaces was the issue of homelessness, which shifted in severity depending largely on the nature of security and policing in each space (Middelmann, 2022a,b). Homelessness in Johannesburg has been driven by a complex mix of brutal, migrant-labour economies, unemployment, poverty, familial and health crises, and a lack of affordable accommodation, all exacerbated by prejudice and violent policing by both private and state actors (*ibid.*). Spaces controlled by private security representing local business interests were often quick to preclude or prevent homeless people settling, whereas in publicly owned and managed spaces (i.e., city departments, local police), homelessness was much more frequent (Middelmann, 2020). And yet, displacement and harassment by police remained very common, showing how circumstances faced by homeless people were disabling *and* debilitating. While homelessness did not start as my core research question, it demonstrated complex answers to two aspects of my investigation. Firstly, for my academic investigation, it revealed critical injustices regarding the interplay between design, history, management and use of public space, suggesting needs for holistic approaches to public space based on wider collaboration. Secondly, in terms of researching the public space of my hometown and my place in it, highlighting disconnection, inequalities and differences, relating to my positionality as a relatively wealthy, university based white researcher working in Johannesburg’s inner city, which is mostly black, largely relatively low-income.

This process of learning about the city and myself,^3^ in line with how my art practice was developing, provided ways of working through some of these inequalities and disjunctures by connecting aspects of my research with my life. Ethically, in conducting research on spatial injustice, I felt compelled to use my life and work, at least in part, to work at addressing those injustices. As I deepened my research engagement with homelessness, including homeless people and various practitioners in the sector, I found opportunities to use my skills and insight to support their work. I began volunteering for the local and national homelessness networks as a researcher,^4^ which developed my understanding of homelessness and thus advancing research. Crucially, this manifested through improving and deepening my connection to people and the processes I was researching, expanding both my understanding as a researcher and impact as a person.

## 3 Homelessness, disability and debility

This paper looks both at the intersection of disability and homelessness as well as looking at homelessness itself through a disability lens. I argue this helps reveal important characteristics and complexities of homelessness, which in turn has implications for understanding, researching and working with homelessness. Among several connections, disability studies shows how homelessness is itself disabling *and* debilitating because of the stigma, harassment, persecution, as well as exclusionary circumstances of society (Mashau and Mangoedi, 2015; Puar, 2017). Mashau and Mangoedi (2015) tie exclusion of homeless people to the history and reality of exclusion during colonialization, apartheid and xenophobia. Critical theory on disability studies—acknowledging complexities of the field, divergences of perspective, and multiplicity and heterogeneity of disability communities—has built on and with feminism, intersectionality, critical race and queer studies to help show that these historical structures of oppression have played significant roles in shaping discrimination as it continues today (Goodley et al., 2019). Schalk (2022) is one recent example of a demonstration of the entanglement of oppressions, focusing as she does on racism and ableism and addressing these issues as connected which is critical for collective liberation. Evoking the importance of intersectionality, discussed more below, this paper builds on arguments to foreground intersectionality more explicitly in research, theory, praxis and advocacy relating to homelessness *and* disability.

One of the disabling impacts of homelessness involves chronic and often undiagnosed illnesses which are relatively common for homeless people. Exclusion faced by homeless people extends to healthcare systems, and is exacerbated by prejudice and discrimination. Furthermore, Mji (2006) shows in the South African context that homelessness makes people disproportionately prone to further disability, partly because of the denial of access to healthcare. Mji (2006: 355–56) points out that the ‘isolation, rejection and marginalization that comes with homelessness is compounded by disability’, noting the cyclical effects of exclusion which show how homelessness is also *debilitating*: ‘poverty makes people more vulnerable to disability, and disability reinforces and deepens poverty.’ Another example of the debilitating circumstances of homelessness that was common during my research in Johannesburg relates to requirements of bureaucratic documentation for accessing basic services and supports. It is exceptionally common for homeless people to be robbed by criminals, or have their belongings ‘confiscated’ by police, thereby exacerbating their denial of access. These multi-directional causal links between poverty, debility, and disability, linked to homelessness, have been noted in many other contexts, including Crawford (2013), warranting further comparative research. More specifically, mental health crises can be involved in causes of as well as being caused by homelessness (Padgett, 2020). Indeed, Martin (2022) has shown how under neoliberalism, people experiencing homelessness and madness are often irresponsibly conflated, highlighting the need for further research into their complex intersections. This speaks to expanding literature on how irresponsible deinstitutionalisation and lack of holistic mental health supports are directly involved in the growth of homelessness (Milaney et al., 2022).

Relatedly, writing like that by Lemus-Mogrovejo (2018) is part of a growing recognition of the intersectional impacts on people who are homeless and have other disabilities. While homelessness is disabling, and the way society treats disability makes disabled people more at risk of homelessness, intersectionality shows how people who are homeless *and* have other disabilities are especially marginalized, exacerbated by being racialized (also noting the impacts of heteronormativity, patriarchy, and other systems of domination and oppression). Intersectionality also helps demonstrate how disability and homelessness are both historically embedded experiences shaped by politics, economics and culture by emphasising how the entanglement of processes combine to produce oppression and marginalization in complex ways across differing contexts. This connects broadly to the strictures of life in neoliberal capitalism and ableism:

‘Neoliberal-ableism is the elision of national economic independence with an individual and cultural celebration of autonomy (Goodley, 2014) [which] … associates happiness with self-reliance. Hence, while people with physical, sensory and cognitive impairments risk experiencing disablism, all individuals of contemporary society are imperilled by the practices of ableism’ (Whitney et al., 2019: 1478.)

Nevertheless, while all are imperilled in these ways—constituting circumstances of widespread debility—intersectionality demonstrates how these constellations of forces impact people differently.

I have written elsewhere about practices and patterns of sharing by residents of Pieter Roos Park, where people exchanged goods and information in support of each other (author…). Mji (2006) discusses how disabled people in a homeless shelter came together around the idea that together challenges can be overcome, despite people having different priorities and challenges. Mji (2006) (*ibid.*: 355) goes on to describe some dynamics that evoke the importance of authentic connection and work across difference: ‘The value attached to interpersonal contact and support between homeless disabled people was matched with the importance accorded interpersonal contact and connection with able-bodied people who may or may not be homeless.’ This work is important in starting to show how homeless people and the issue of homelessness can teach us not only about how to research, but critical lessons on how to *be* as people.

## 4 Self/other/collective: public culture and the threshold of privacy

**Figure 4:**
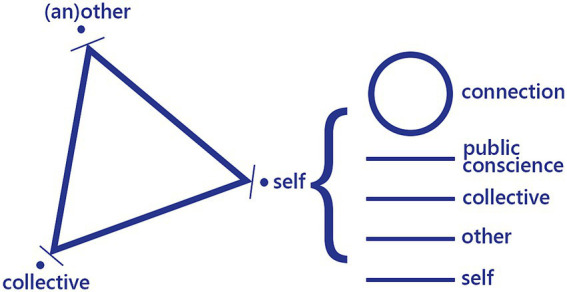
Diagram of self-other-collective triad, produced by author.

This section explores my attempts to remain open to how identities are formed and constituted mutually through interaction Erwin (2012), suggesting that these processes both reflect and shape public culture. This is demonstrated through a triad that signifies relationships between individuals, other individuals, and collectives (see Figure 4 below). Understood through this triad, and pursued through my research and artwork, I have tried to be open to how interlocking identities are constructed (and potentially transformed) through interactions in public space (Ruddick, 1996). Instead of approaching a conversation with ideas around who ‘I’ am and who ‘you’ are, I aimed at approaching a conversation as openly as possible, looking at the space between ‘you’ and ‘I’. Simone (2021: 1347) explores how this is necessarily open-ended: ‘Something is apportioned out to us as we apportion ourselves out to it, in a process of mutual figuring rather than the imposition of our intentions upon the objects or experience within the environment … set against a backdrop where there could be many different alternative realizations.’

Landman (2019: 120) suggests that in South Africa, these dynamics are still rooted in the history of colonialism and apartheid, which created an “‘us’ and ‘them’ syndrome that is still prevalent.” This implies a long-term culture of disconnection and division in Johannesburg and other South African cities, connecting to growing polarisation globally (Carothers and O’Donohue, 2019) and to tensions within potential communities of care and wellbeing (e.g., Runswick-Cole, 2014). This necessitates inclusive politics to counter the neoliberal preoccupation ‘with defining and maintaining the borderlands between “us” and “them”’ which sustains polarization and excludes those not corresponding to the ‘ideal neoliberal type’ (Runswick-Cole, 2014: 1124, citing Ramlow, 2006). While South Africans are often guided into interactions in public places by prejudice and this public culture of disconnection, I have aimed to avoid predetermining the nature of the interaction and those it involves by focussing on the space in-between the self and another, which is where both ‘you’ and ‘I’ actually take their meanings in the moment. Notwithstanding moments where I have fallen into patterns of retreat, prejudice, or fear, focus on the threshold between public and private can bring new prospects for the relationships between selves and others starting from the micro-level (Whaley, 2018). This is the space where we work out who we are, who others are, and thus where publics are formed. Therefore, these moments are part of how public culture is shaped, reflecting Zukin’s (1995: 11) description of “[p]ublic culture as socially constructed on the micro-level.”

In my research methods and associated public art practices, I continue aiming towards reducing the boundary between self and other, between the internal and external worlds. Deepening this autoethnographic reflection and practice connects me with De Beer’s (2013: 1) exploration on connections and disconnections between the self, others, the university, the city, and its homeless residents: ‘carefully and deliberately seeking to hold on to different publics, seeking ways not to have to exchange one world of immersion for another, struggling between presence and absence, feeling slightly dismembered and trying to make sense of it’. My research and art are both important here, because without their intersection, these shifts would not have taken hold, at least not in these same ways. Many of course have developed more engaged, collaborative research practices without an associated art practice. However, my art practice and the ways it changed me have been central to these shifts, and the insights are relevant in other contexts, especially amidst calls for more community-engaged, social-impact oriented research. In exploring how to manifest this for myself, a core goal of mine is to develop an associated art and research practice that corresponds to de Certeau’s (1980/1984: 110) exploration of how spatial practice can incorporate ‘the joyful and silent experience of childhood; it is, in a place, *to be other and to move toward the other*’. This paper explores how this necessitates an apparent sacrifice of self, but one that allows a more authentic self to emerge in and through community.

A key learning from my fieldwork and art practice is that breaking down the barrier between self and other requires foregoing some private, internal space (ego). The boundaries between the self and others are permeable and blurred (Whaley, 2018). Following the assertion of this permeability, Whaley (2018) argues for a wider understanding of self, where the self can tend towards inclusion of others. I argue that Whaley’s (2018) reconceptualization of the self as inclusive, sacrificing some attachment to pure individuality, allows for the boundary between self and other to be reduced, which in turn opens space for the formation of collectives through a culture of inclusion. This does not deny space for difference nor even contestation. In fact, Whaley (2018: 32) argues that in the context of a more inclusive vision of self, ‘[d]iversity and difference are understood not as threats or challenges but as vital sources of sustenance and enrichment.’ As such, the sacrifice of self is only apparent; one can become more fully oneself by connecting with others. While entering a public space as a researcher and outsider emphasised division, when I began entering public space to offer my art practice, one of my blessed gifts, I was able to be fully and authentically myself while also moving towards others.

Some of these moments, or interactions, are more revealing when looked at through the graphically represented symbolic relationship between the self, (an)other and the public, shown by the triangle on the diagram’s left side. The points on the triangle constantly shift and morph according to space, time, energy and actions. In other words, the shifting points of the triangle refer to connections, entanglements, and ‘transitions between the public and the private’ (Lefebvre, 1992/2004: 95). Despite each point remaining partly distinct from each other and the triangle, the self, others, and the public are necessarily mutually constitutive. The points connect directly in moments of interaction, which have a charged potential to shift the relationship between them. When the self and the other (self and another, or others) become closer towards each other, a wider public can become more concretely constituted, generating inclusion on a micro-level. This requires openness to and celebration of difference, which relates to a call by Mbembe (2018) to break down barriers between self and other, both practically and philosophically. When the self and others are pushed further apart (physically, spatially, emotionally, psychologically), formation of publics becomes more fractured and tenuous, allowing narrow interests to prevail. Whaley (2018: 30) argues that this fracturing often stems from fear and a view of the self and other as “mutually exclusive,” which can tend towards a public “culture of exclusion,” undermining both publicness and attempts towards spatial justice.

The right-hand part of the diagram disambiguates elements of the self, from the first pole of the self, the second pole representing another, the third pole of collectives, and the fourth of public conscience. The circle above shows that these different levels are connected for everyone, reminding us that the different levels, ourselves and others, are all part of the same whole. Importantly, the triangle is whole, demonstrating the reality of entanglement and mutual constitution between selves, others and collectives. Furthermore, implicit is the constellation of these relationships creating webs of interaction starting from all selves and constituting collectives through interpersonal contact. As written by de Certeau (1980/1984: xi), ‘each individual is a locus in which an incoherent (and often contradictory) plurality of such relational determinations interact’, noting how these ‘systems of operational combination … also compose a “culture.”’ As people move towards and with one another, this culture becomes more public, more inclusive, and more supporting of communities of wellbeing.

Analysis through this triad aims to build on ‘disability studies scholarship today [which] shows how disability troubles normative concepts of self, other, agency, labor, property, and relationality’ (Kim et al., 2021). All three points on the diagram are entities, but each is entangled and overlapping with, and as such mutually constitutive of the other two. One cannot be a ‘self’ without there also being people who are not the ‘self’ but are ‘another’. Moreover, neither the self nor the other can exist without being part of a greater whole: a collective which corresponds to a/the public. This relates to the principle of *ubuntu,* ‘which some philosophers consider to be a quintessentially African notion of interdependence (‘a person is a person because of other people’). Ubuntu establishes clearly the virtue of care in its strongest sense’ (Hassim, 2022: 60). This principle—in holding the 3 points on the diagram together—guided my attempts to break up the public-private binary during my research; our private spaces exist within public space. Our self (the smallest unit of private space) encounters all others (in their private spaces of the self) in space that is external to both the self and another: public space. There is a specific overlap and complexity here relating to homelessness in how private behaviours are forced into public space, creating a tense threshold between public and private. Erwin (2012) argues that these encounters, whether tending towards inclusion or exclusion, are where our individual identities are formed, implying that such encounters shape the formation (or potentially division) of publics and public culture. Thinking through my research in this manner correlated with attempts to bring my work and life closer together (Morreira, 2012).

## 5 Curiosity, art, advocacy: towards inclusive publics

I am grateful that I have found ways to make my research and art practice more outward-facing, connected, and engaged. While the way I frame and understand the triad suggests urges towards collaboration and connection across difference, critical disability theory and praxis of interdependence brings this all together. Interdependence and creative resilience/resourcefulness is also something that is central to disability studies and that I have observed in my research on homelessness. Interdependence requires adapting to the needs of others, i.e., moving the self towards others in the service of an inclusive public. Exploring the social model of disability through the lens of dementia, Milligan and Thomas (2016: 13, 14) speak about the importance of collaboration in understanding ‘relationship between the individual and community … understanding and valuing difference can only be resolved through the engagement of all involved.’ Critical disability studies has done a lot of important work in shifting from individualisation towards solidarity and interdependence, with important lessons for researching and addressing homelessness by working across difference. In a review of Piepzna-Samarasinha’s (2018) *Care Work,* Whiddington-Sadlowski (2023) articulates this shift: ‘Instead of presenting her disability as an “illness that she overcame,” she wrote about collective struggle and community building.’

Looking inward is part of the beginning of moving towards others. Important in driving this is curiosity, wonder, and play. Curiosity is linked to an appreciation of mystery that has been central to my art practice that aims to get deeply into the space of the unknown; ‘to eliminate the unforeseen or expel it from calculation as an illegitimate accident and an obstacle to rationality is to interdict the possibility of a living and “mythical” practice of the city’ (de Certeau, 1980/1984: 203). I argue, partly in following Paula Toledo, that this openness to mystery is related to an openness to difference which is foundational for collaboration. Toledo (2018) explores links between curiosity and openness to mystery, suggesting these as important bases for getting to know others from a place of compassion. Connecting strongly to the disability justice principle of ‘meeting people where they are’ (e.g., Choi, 2021), this compassion can in turn build connection, gratitude, and openness to wonder which keeps curiosity alive. This also links with the personal curiosities and connections that bring us into conducting research in any field or context. I argue that foregrounding these personal connections more openly and honestly, rather than insisting on scientific objectivity, is important for practising more inclusive research, especially if considering research methodologies as part of developing inclusion more widely. An important dimension of the methodological implications of these arguments pertains to responsibilization of the individual, which is part of the individualism critiqued and explored earlier in this paper. The key idea here is that while this paper suggests internal shifts as part of the core of opening to inclusive publics, that the possibilities, especially for junior researchers in highly competitive, neoliberal institutions, are constrained by time-limits, funding requirements, expectation of continuous, high-level output. As such, a crucial avenue for future research is about how institutions and academia can change to accommodate these sorts of shifts.

Ultimately, my guiding impulse that resonates with artist and researcher Rosie Priest’s (2020: unpaginated) call for collaborative practice across difference is to ‘work collectively with the spaces of marginalization, not to erode them and bring them into the middle, but rather to celebrate and support, to explore and learn from’ which necessitates ‘long-term meaningful work with the communities whose voices, experiences and creativity have largely been neglected by the mainstream.’ This kind of long-term work in research, spending open-ended time with people, connects to Riles’s (2022 unpaginated) work on play and playfulness: ‘Scholars need space for open, free form wondering and tinkering.’ This playfulness and creative approach to life is a key link between my research and creative practices. Because there are multiple, inter-related, highly complex problems, creative collaboration is important in unearthing holistic solutions. Indeed, Schwan et al. (2018) have explored and worked with the power of art to support homeless youth in managing their stress, healing from trauma, telling their (otherwise often silenced) stories, and breaking down barriers with law enforcement and health authorities. Essentially, while my reflections on art, research and life are not intended as a necessarily replicable research tool, this type of work demonstrates how play, creativity, and art can be powerful in addressing complex, multi-layered issues that unsettle the distinction between work and life.

My art practice and advocacy work in the homelessness sector have generally not been an ‘official’ part of my academic research, not featuring in my research proposals, funding applications, or ethics procedures through the university; they were not directly part of my data collection that were analysed alongside observational, interview and other data. However, this is why the paper explores how research and life are related: because living my life and deepening connection to the research context has been an important process for my attempts at practicing inclusion and working for justice, *including in my research practice.* As such, this work at the edges of my research has played fundamental roles in shaping both my understanding of the research context and topic, and importantly in shaping my relationship with the issues I’m researching. This manifests through my art in moving myself and my praxis towards community, and in my advocacy through shifting my research from more extractive and individualistic, to more collaborative, action-oriented and activist.

Importantly, moving towards forming and sustaining inclusive collectives and publics does not fully address the tensions, domination and violence that can and does manifest between publics. As such, moving towards deeper and more inclusive forms of ‘collective life … does not obviate the ways collectives will need to deliberate and negotiate questions about what they want to be and how to live together.’ (Simone, 2021: 1348). In working against the individualisation of neoliberal society, Nishida (2022) introduces the idea of affective collectivity, which calls to centre human connection, solidarity and care. Further research is required about the relationships between collectives, how to address growing polarization, and beyond the shifts explored in this paper, what is required to develop a broader, mutual solidarity across various forms of difference. While this paper is limited by my mostly South Africa-based experience of researching homelessness and creative practice, it is informed by global and especially Canadian literature on homelessness and disability.^5^ This highlights the need for further research across contexts, linked to the need for research and action across difference and discipline, within and beyond the research context.

## 6 Conclusion

The paper largely focuses on the conditions under which we carry out our research methodologies, and the kinds of shifts that undergird methodologies that can support more inclusive publics and communities of care. Despite focusing on these political, spiritual, and ethical matters, some clear methodological implications also emerge. The importance of play is linked to curiosity, wonder, and openness—I argue that making time for creative, playful approaches as part of our methodological toolkits is important in foregrounding authentic relationships between researchers and participants. The issue of time for play extends to other aspects of the research process. Spending honest, open-ended time with people and in the context of research can deepen understanding and appreciation of multiple perspectives and complexity. In other words, slowing down, partly inspired by Piepzna-Samarasinha’s (2019) “revolutionary slowness,” allows for greater reflexivity and expansive work across difference. Reflexivity is key to how internal shifts towards and with others can be better understood and consolidated, and for being more open and transparent about our reasons for and connections to research on the topics, themes or people we worth with.

In exploring the conditions and circumstances of research and associated methods, the paper uses a triad to examine the relationship of individuals (selves, private space) and others, exploring the implications of how these relationships constitute collectives (publics). To do so, in aiming to explore how this triad relates to research methodology, the paper uses two lenses. Firstly, the authors merging art and research practices as driving forms of curiosity, play, and openness to difference, including shifts towards homelessness advocacy beyond research practice. Secondly, using the lens of homelessness to explore aspects of these relationships in a research context, exploring how homeless people and others in the sector worked towards developing cross-cutting communities of care, and crucially working across difference. These are argued to be critical for broader, more inclusive publics and as such, communities of mutual support and wellbeing, as evidenced by realities of homelessness viewed through a critical disability lens. In contexts of inequality and researching marginalized groups, especially for relatively privileged researchers, openness about complicity in the conditions of marginality and inequality is an important part of honest connection across difference.

## Data Availability

The datasets presented in this article are not readily available because the consent process did not make allowance for data to be shared outside of use by the researcher. Requests to access the datasets should be directed to Temba Middelmann, tjdm90@gmail.com.

## Footnotes

^1^There remains tension between perspectives on terminology for homeless people, which I have referred elsewhere in this paper to as public dwellers (and in Middelmann, 2022b), reflecting some reality of their living situation. Terms like unhoused help to start specifying some complexity of the multiple types, causes and consequences of homelessness. This paper uses different terms according to context, also aiming to connect to multiple discourses that may each employ different language.

^2^And beings, and space, and energy, and rocks and plants, though there is not space to deal with these in this article.

^3^A paper with a focus on positionality, language, reflexive research methods, complicity and global inequality has been recently accepted for publication in another journal.

^4^And later, as a treasurer. While this was not my main expertise, this was one of the needs of the community. Sidelining my ego to some extent allowed me to contribute in this way.

^5^Noting that the author has been living and working in Canada since 2023. My experiences in these two contexts suggest resonance regarding the dynamics discussed in this paper, though further collaborative and comparative research is required.
